# The critically endangered forest owlet *Heteroglaux blewitti* is nested within the currently recognized *Athene* clade: A century-old debate addressed

**DOI:** 10.1371/journal.pone.0192359

**Published:** 2018-02-05

**Authors:** Pankaj Koparde, Prachi Mehta, Sushma Reddy, Uma Ramakrishnan, Shomita Mukherjee, V. V. Robin

**Affiliations:** 1 Division of Conservation Biology, Sálim Ali Centre for Ornithology & Natural History, Coimbatore, Tamil Nadu, India; 2 Manipal Academy of Higher Education, Madhav Nagar, Manipal, Karnataka, India; 3 Indian Institute of Science Education and Research, Tirupati, Andhra Pradesh, India; 4 Wildlife Research and Conservation Society, Pune, Maharashtra, India; 5 Loyola University, Chicago, Illinois, United States of America; 6 National Centre for Biological Science, TIFR, Bangalore, Karnataka, India; Leibniz-Institute of Freshwater Ecology and Inland Fisheries, GERMANY

## Abstract

Range-restricted species generally have specific niche requirements and may often have unique evolutionary histories. Unfortunately, many of these species severely lack basic research, resulting in poor conservation strategies. The phylogenetic relationship of the Critically Endangered Forest Owlet *Heteroglaux blewitti* has been the subject of a century-old debate. The current classifications based on non-phylogenetic comparisons of morphology place the small owls of Asia into three genera, namely, *Athene*, *Glaucidium*, and *Heteroglaux*. Based on morphological and anatomical data, *H*. *blewitti* has been alternatively hypothesized to belong within *Athene*, *Glaucidium*, or its own monotypic genus *Heteroglaux*. To test these competing hypotheses, we sequenced six loci (~4300 bp data) and performed phylogenetic analyses of owlets. Mitochondrial and nuclear trees were not congruent in their placement of *H*. *blewitti*. However, both mitochondrial and nuclear combined datasets showed strong statistical support with high maximum likelihood bootstrap (>/ = 90) and Bayesian posterior probability values (>/ = 0.98) for *H*. *blewitti* being nested in the currently recognized *Athene* group, but not sister to Indian *A*. *brama*. The divergence of *H*. *blewitti* from its sister taxa was between 4.3 and 5.7 Ma coinciding with a period of drastic climatic changes in the Indian subcontinent. This study presented the first genetic analysis of *H*. *blewitti*, a Critically Endangered species, and addressed the long debate on the relationships of the *Athene*-*Heteroglaux*-*Glaucidium* complex. We recommend further studies with more data and complete taxon sampling to understand the biogeography of Indian *Athene* species.

## Introduction

Endemic and endangered species are often ecologically specialized and there is an urgent need to better understand the ecology and phylogenetic history of endangered species to uncover features that might be crucial for conservation. Such species could be viewed as model species to understand evolutionary processes in the landscape of their occurrence [1–3]. However, our knowledge of the evolutionary history of highly restricted, endemic species in the tropics is limited, due to their rarity and incomplete genetic sampling [4]. This could have far-reaching consequences on policy level decisions regarding species conservation.

Although the phylogeny of the higher order avian taxa has undergone several changes in the past three decades [5–7], relationships among clades still remain poorly known. Owls (Order: Strigiformes) is one such groups. Many rare or range-restricted species such as the Critically Endangered Forest Owlet *Heteroglaux blewitti* (Hume, 1873), Spotted Owlet *Athene brama* (Temmink, 1821) and Jungle Owlet *Glaucidium radiatum* (Tickell, 1833) were not included in the most recently published owl phylogenies [8–10].

*H*. *blewitti*, endemic to India, has been a taxonomic mystery since its discovery in 1872. Owing to its severely fragmented distribution and low population, *H*. *blewitti* has been categorized as a “Critically Endangered” species by the International Union for Conservation of Nature (IUCN) [11]. There have been many opinions on the phylogenetic affinities of *H*. *blewitti* by ornithologists over the centuries. In the past, the species has alternatively been placed in either the genus *Heteroglaux* [12–16] or *Athene* [17–22]. Additionally, some researchers have claimed *Heteroglaux* to be a subgenus of *Glaucidium* [23], related to the tail-flicking behavior typical in the genus *Glaucidium*. Nevertheless, none of these opinions were founded on formal phylogenetic analyses.

The genus *Athene* is represented by four species—Burrowing Owl *A*. *cunicularia*, Spotted Owlet *A*. *brama*, Little Owl *A*. *noctua* and White-browed Owl *A*. *superciliaris* [9, 15]. All the extant *Athene* members were classified in the genus *Strix* when first described. Following a revision in taxonomy [17], *A*. *brama* and *A*. *noctua* were placed in the genus *Athene*, a placement that remains unchanged to date. *A*. *cunicularia* was moved from *Strix* to *Speotyto* [24] based on DNA-DNA hybridization studies, and later to *Athene* [8], based on mitochondrial CYTB and nuclear RAG-1 gene data. Similarly, *A*. *superciliaris* was moved from *Strix* to *Ninox* [25], and then to *Athene* [9]. Throughout this article, we refer *A*. *brama* and *A*. *noctua* as Eurasian *Athene* (with global distribution encompassing India) and *A*. *superciliaris* and *A*. *cunicularia* as *Athene* from Madagascar and the Americas.

**Fig 1:**
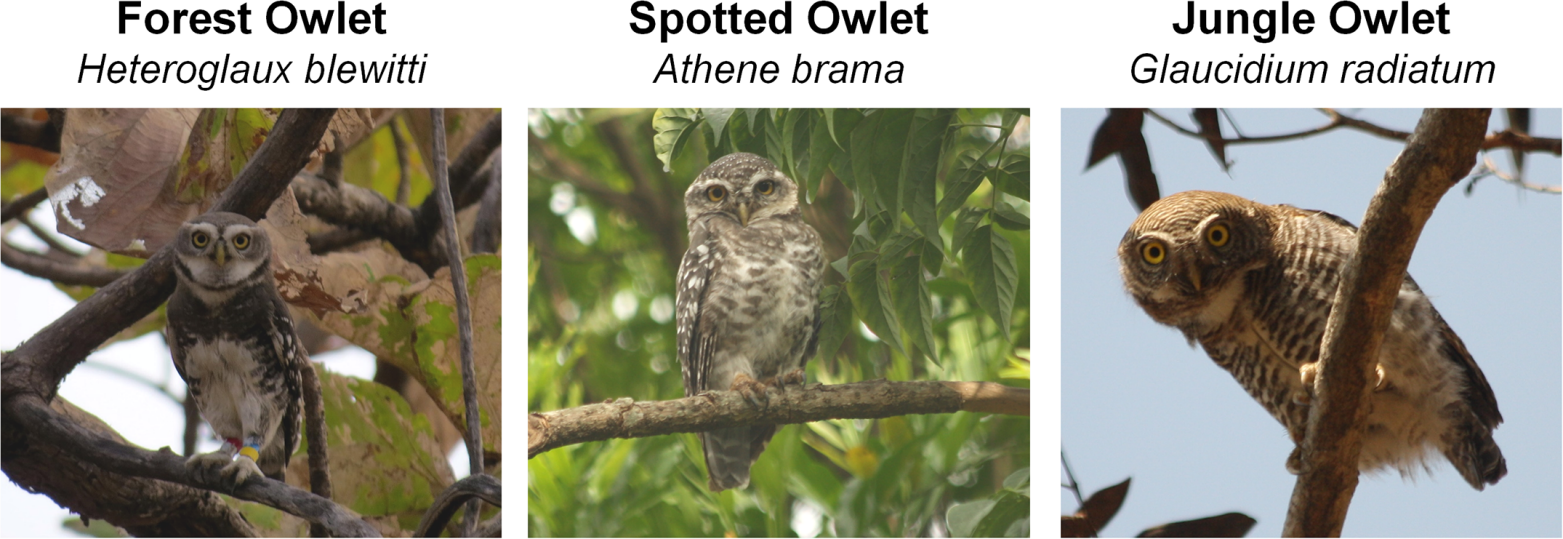
Co-distributed Indian owlets show plumage similarity, however can be identified based on size and markings on the chest and forehead. Presence of white spots and brown bars in case of *A*. *brama* and *G*. *radiatum* respectively are identification keys. Photo credits: color banded *H*. *blewitti* individual by PM, *A*. *brama* and *G*. *radiatum* by PK.

Since *H*. *blewitti* and *A*. *brama* are morphologically similar in appearance [17] and are co-distributed (Fig 1, S1 Fig), they would be expected to form a sister group. Although Wolters (1975) hypothesized that *H*. *blewitti* and *A*. *brama* together form a subgenus *Heteroglaux*, nested within *Athene* [26], he did not provide an explanation for this classification [16]. In contrast, König *et al*. (1999) argue that the tail flicking behavior, a characteristic of *Glaucidium*, shown by *H*. *blewitti*, suggests that the species is closely related to *Glaucidium* and could be nested within *Athene* or *Glaucidium* [23]. The current classification of *H*. *blewitti* in a monotypic genus *Heteroglaux* claimed by Rasmussen & Collar (2013) is based solely on an assessment of morphological [12, 16] and osteological characteristics [16], without phylogenetic data. This classification needs further scrutiny by incorporating phylogenetic information. Furthermore, a report on the hybridization between *H*. *blewitti* and *A*. *brama* [27], was disputed [28–31], and this underscores the need to examine the taxonomic status of the species. Data available on acoustic [32], morphological, osteological and behavioral characters [16] of *H*. *blewitti* show that the species differs from other *Athene* species in osteological features such as multiple cranial characters, especially, wider, inflated anterior and posterior frontals, larger lacrimals and maxillopalatines, stouter tarsometatarsi, and behavioral features such as non-undulating flight and tail flicking. Rasmussen & Collar (2013) argue that this difference could well be at the genus level [16]. The authors further propose that the plumage similarities in *A*. *brama* and *H*. *blewitti* could be due to convergence but given the distinct osteological and behavioral features of *H*. *blewitii*, another possibility is that *H*. *blewitti* evolved from an ancient divergence event separating the genus *Heteroglaux* from *Athene* [16].

We test three proposed phylogenetic relationships as competing hypotheses—Rasmussen & Collar (2013) [16], König *et al*. (1999) [23], and Wolters (1975) [26] using molecular data to infer phylogenetic relationships among *H*. *blewitti*, *A*. *brama*, and *G*. *radiatum*. Our study will also address the debate about *Athene*-*Glaucidium*-*Heteroglaux* relationships using genetic data. We also expect that this new phylogenetic information on an endemic and Critically Endangered species will help understand priorities in conservation strategies.

## Materials and methods

### 1. Taxon sampling

Based on data from extant phylogenies [8–10], we generated data on the three Indian Owlets *H*. *blewitti*, *A*. *brama*, and *G*. *radiatum* as well as the Madagascan species *A*. *superciliaris*. We sampled, three out of five subspecies of *A*. *brama* namely *A*. *b*. *brama* (North India), *A*. *b*. *indica* (South India) and *A*. *b*. *albida* (Parts of Gujarat, Pakistan, and Iran), and two subspecies of *G*. *radiatum* namely *G*. *r*. *radiatum* (Peninsular India) and *G*. *r*. *malabaricum* (Southwest India). The assignment of subspecies was based on distributional limits described in Ali & Ripley (1983) [19]. For field-based sample collection from the three species of owls (*H*. *blewitti*, *A*. *brama*, and *G*. *radiatum*), we followed all legalities and obtained prior permissions from State Forest Departments (Madhya Pradesh, Maharashtra, Gujarat and Chhattisgarh). The Madhya Pradesh Forest Department granted one of the authors (PM) permits to capture and color tag *H*. *blewitii* individuals as part of an independent study on the species. The Chhattisgarh Forest Department permitted capture and blood collection, whereas our permits from Maharashtra and Gujarat were limited to visual surveying of the Forest Owlet (*H*. *blewitti*). We captured Forest Owlets using Bal-chatri traps, known to be the most effective trapping technique for capturing birds of prey without inflicting injury [33]. The capture and release protocol was reviewed by the Madhya Pradesh State Forest Department’s expert committee before granting the research permit to PM.

For this study, during the capture process, we handled captured owlets for a maximum of twelve minutes and released them immediately after banding and biometrics procedure. We carried out the banding procedure very close to where the owlet was captured so we could release it at the same spot. Once captured, we covered the head of the owlet with a cloth to minimize stress. We collected feathers that were shed during the process of capture and handling from each bird. We collected up to two feathers per individual. We stored the feathers in separate paper envelopes and placed these in airtight containers for transport. Color tagging of birds ensured that we could identify different individuals and sources for the samples. We used these feathers to create reference genetic data for the species. In two instances, we collected broken eggshells fallen below known nest-sites of *H*. *blewitti*. We compared genetic data collected from eggshells (S1 Table) with the reference genetic data to identify species. We identified the species using a criterion of ≥99% sequence identity with the reference data. After species assignment, we proceeded with further analysis. We also obtained a museum feather sample of *H*. *blewitti* from the Bombay Natural History Society (BNHS) and used the same criteria mentioned above to ensure species identity. In case of *A*. *brama* and *G*. *radiatum*, we collected fallen feathers below known nest-sites outside Protected Areas or shed feathers from injured bird rescued by NGOs. We trapped *G*. *radiatum* individuals in mist nets (in Chhattisgarh). We sampled spatially non-overlapping and distant nest-sites to avoid resampling the same individuals. For both the species, for ensuring species identity, we followed the same approach as for *H*. *blewitti*. We sequenced target genes from a vouchered tissue of *A*. *superciliaris* obtained from the Field Museum of Natural History (details of samples and sources are provided in S1 Table and S2 Fig).

### 2. Laboratory procedure

We extracted DNA using DNeasy blood and tissue kit (Qiagen, Hilden, Germany, Product no. 69504), following the manufacturer’s protocol with a few modifications. We added 20 μl of 1% Dithiothreitol (DTT) in the lysis reaction. To ensure a higher concentration of DNA and minimize loss, we eluted DNA twice in separate vials, each time in 100 μl of AE buffer, instead of the recommended one elute of 200 ul and used the first elute for further analysis. We amplified two mitochondrial genes (CYTB, COI), a nuclear exon (RAG-1), and three nuclear introns (TGFB2, LDH, MYO). These genes were chosen based on previously available data on other species of *Athene* and *Glaucidium* [8, 9]. We carried out all PCR amplifications after optimizations (S2 Table) of reaction conditions. We sequenced the purified PCR products in both forward and reverse directions with an ABI 3730 Genetic Analyzer and analyzed raw sequences with the ABI 3730 Genetic Analyzer software (Applied Biosystems, Foster City, USA). We designed two owlet specific primers for amplifying COI gene from *A*. *brama* and *G*. *radiatum*. We used primers for other genes available from published studies [34–42]. We submitted all the sequences from the study to GenBank (see S3 Table for accession numbers).

### 3. Sequence analyses and phylogenetic reconstruction

We viewed and manually edited the sequences in Chromas Lite 2.1.1 (Technelysium, Brisbane, Australia) and aligned them using the software Geneious v7.0.6 (Biomatters, Auckland, New Zealand) [43]. We downloaded sequences of other owls from GenBank (S4 Table). We translated coding sequences in Geneious to check for the presence of stop codons and/or nuclear inserts of mitochondrial DNA (numts). We processed individual gene alignments in MEGA v4.0 [44] for counting the proportion of variable sites, parsimony informative sites, and singletons. We tested for positive selection, to avoid introducing possible error in phylogenetic inference as shown in [45, 46], in CYTB, COI, and RAG-1, using HyPhy [47] and Tajima’s test of neutrality [48] implemented in MEGA v4.0.

We conducted the analyses using three different sets of data—mitochondrial (CYTB + COI), nuclear (RAG1 + TGFB2 + MYO), and concatenated (CYTB + COI + RAG1 + TGFB2 + MYO). There was a missing in-group taxon (*A*. *noctua*) in the LDH dataset; therefore, we did not include the dataset in the final combined analysis. The concatenated dataset contained <9% missing data. We used codon-specific DNA substitution models (PartitionFinder v1.1.1, S5 Table) [49]. We tested the separate gene as well as concatenated datasets for best-fit DNA substitution models (Details in S5 Table).

We conducted phylogenetic analysis with maximum likelihood using RaxML v8.0 [50], Bayesian Inference using MrBayes v3.2.2 [51], and multi-species coalescent tree using BEAST [52]. We used members of the Tytonidae family (*Tyto alba* and *Phodilus badius*) as outgroup taxa since this is the family closest to the Strigidae family with an estimated known divergence time for the Strigidae / Tytonidae split (54–83 Ma) [53–56].

We used PartitionFinder to first determine the best partitioning scheme of gene regions based on evolutionary rates. We used these partitions in RAxML and MrBayes. In RAxML, we used ML+rapid bootstrap function with 10000 bootstraps for all analyses. In MrBayes, we conducted two runs of five chains (one cold) for 30–70 million generations and sampling every 1000th generation. We set the temperatures of the heated chains to 0.25. We discarded the first 25% of samples (burnin) and continued the MCMC run till the standard deviation of split frequency dropped below 0.005.

We ran each ML and Bayesian analysis thrice, to ensure consistency in the results, for the concatenated dataset with the following options—partitioning of the dataset in all codon positions of coding sequences, only the third codon position of coding sequences, and all codon positions for the mitochondrial genes and only the third codon position for the nuclear exon.

We used the concatenated dataset, without LDH data, to build species phylogeny in Beast v1.8.1. The species tree analysis does not take into consideration columns with missing data; hence, we did not include LDH dataset for which *A*. *noctua* data was missing. We ran the analysis for 1.5 billion runs. We viewed and edited the trees in FigTree v1.4.2. We also used DensiTree v2.2.5 [57], based on the Bayesian output of BEAST, to plot sets of trees.

To test for congruency in mitochondrial and nuclear datasets, we performed a Shimodaira-Hasegawa test [58] in RaxML. We also conducted gene jack-knifing analysis in which we serially removed individual genes from the concatenated dataset to see which gene/s may influence the phylogenetic analysis [39].

### 4. Fossil calibrations and molecular dating

Owls have an adequate fossil and sub-fossil record, largely from Europe and North America [59]; however, classification of many of the sub-fossils remains ambiguous [60]. In addition, very few phylogenetic studies of Strigiformes have used molecular dating and there is no consistency in fossil calibrations used. Only fossil calibrations with sufficient support, as discussed in [61, 62], and those that have been used in multiple studies, were used in this study. We used *A*. *otus* / *O*. *leucotis* (23.7–16.4 Ma) [63] and the oldest *Athene* fossil (3.6–5.3 Ma) [64] for calibrations. We used different combinations of data (concatenated, mitochondrial, and nuclear, partitioned alignments), to check for consistency in results, to obtain molecular dates after performing tests for a molecular clock [65] in MEGA v4.0 and using both the strict and the uncorrelated relaxed lognormal clocks. Substitution models, clock models and trees option were set to unlinked for all the partitions. We used the lognormal distribution for fossil calibrations with the means of distributions set such that 95% of the distribution probability fell within expected ranges of time intervals. We ran BEAST on CIPRES portal (www.cipres.org) for 1 to 2 billion MCMC runs. We set up the sampling frequency at 1000 and re-sampled data using Log Combiner v1.8.1. The BEAST output was viewed in Tracer v1.6 and trees were combined in TreeAnnotator v1.8.1. We compared our results with other studies to check for consistency of our molecular date estimates. We first compared our results with Fuchs et al. (2008) [66] who used Mlíkovský (1998) fossils of *A*. *otus* and *O*. *leucotis* [63], along with a geographical event dating which does not include our study area. Further, we compared our Strigidae / Tytonidae split dates with other relevant studies [53–56]. We used Effective Sample Size (ESS) values as one of the criteria to compare among analyses.

## Results

### 1. Phylogenetic analysis

**Fig 2:**
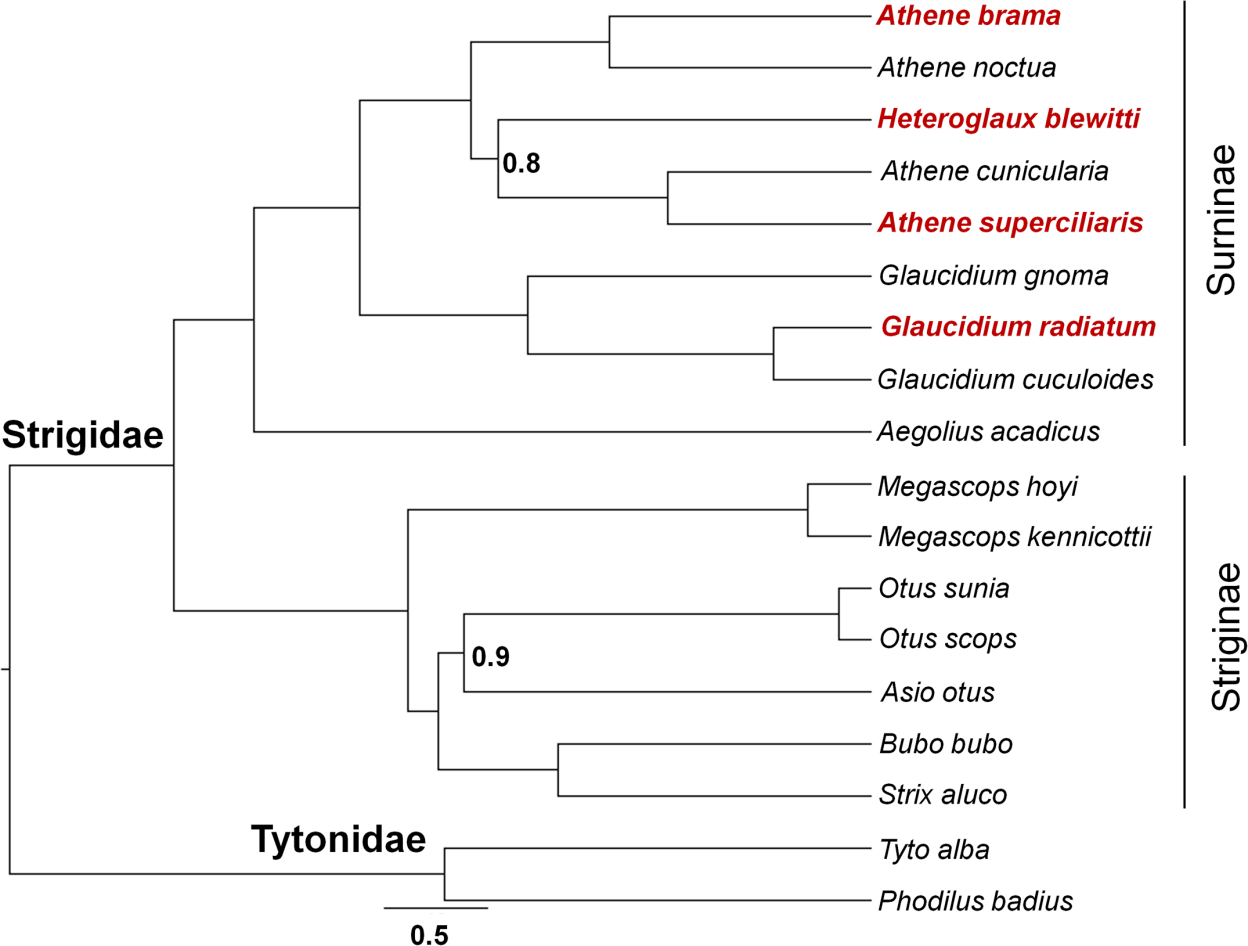
A species tree reconstruction using BEAST on concatenated (mitochondrial + nuclear) dataset indicate that *H*. *blewitti* is nested within the *Athene* clade. The brown text indicates the species sampled in the present study. The nodal values show Bayesian posterior probability (PP). All the nodes are highly supported (PP = 1) except for those where PP is mentioned as nodal value.

**Fig 3:**
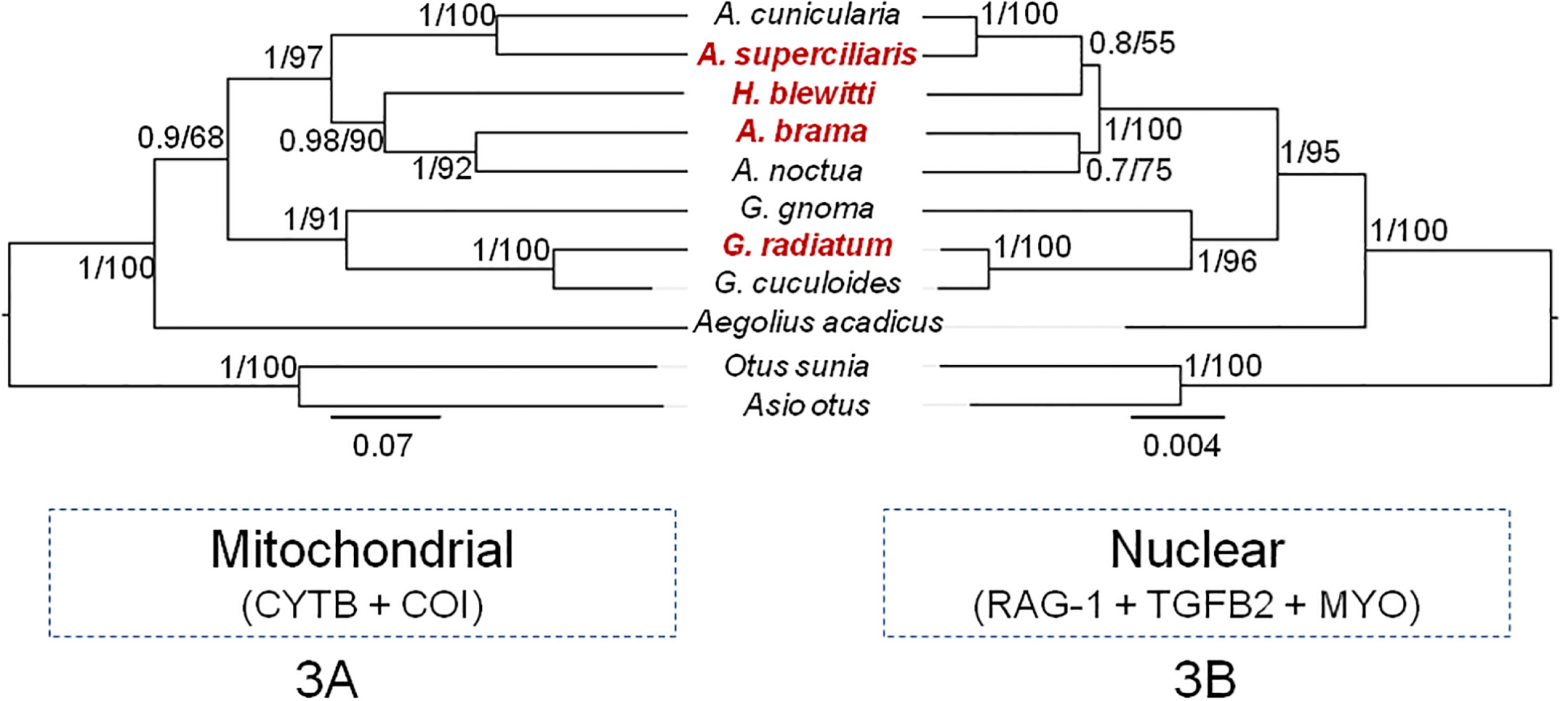
A Maximum Likelihood Phylogenetic tree of *Athene*-*Heteroglaux*-*Glaucidium* members. **3A**: Tree constructed using mitochondrial (CYTB + COI) dataset; **3B**: Tree constructed using nuclear (RAG-1 + TGFB2 + MYO) dataset. The red text indicates the species sampled in the present study. The nodal values indicate Bayesian posterior probability separated by maximum likelihood bootstrap support.

In the concatenated tree analysis, we recovered *H*. *blewitti* as nested within the *Athene*, and sister to the other *Athene* from Madagascar and Americas (Fig 2 and S3 Fig). We observed that in all gene trees (S4–S9 Figs), *A*. *brama* and *A*. *noctua* were sisters to each other. Similarly, *G*. *radiatum* and *G*. *cuculoides* were sisters in all the analyses. We did not find significant congruence (*P* < 0.01) at the *H*. *blewitti* node in mitochondrial (S10 Fig) and nuclear (S11 Fig) trees when we performed the Shimodaira-Hasegawa test (Fig 3 and S3 Fig). In the mitochondrial tree, *H*. *blewitti* was sister to the Eurasian *Athene* clade, whereas in the nuclear tree it was sister to *Athene* from Madagascar and the Americas (Fig 3). We always recovered the mitochondrial tree topology when any one of the three nuclear genes (TGFB2, MYO and RAG-1) were removed from the concatenated dataset during gene jack-knifing.

### 2. Molecular dating analysis

The molecular dating analyses resulted in varied estimates of divergence (Table 1). Our mitochondrial and concatenated datasets, however, failed to converge in dating runs. Based on the analyses for the nuclear dataset (Table 1, analysis 2), for which ESS values were the highest and the Tytonidae / Strigidae and *A*. *otus* / *O*. *leucotis* divergence estimates matched with other published estimates, we predicted diversification dates for *H*. *blewitti* between 4.3 and 5.7 Ma, *A*. *brama* / *A*. *noctua* split between 3.9 and 5.8 Ma, *A*. *cunicularia* / *A*. *superciliaris* split between 2.2 and 3.9 Ma and *G*. *radiatum* / *G*. *cuculoides* split between 0.1 and 1.8 Ma.

**Table 1:** Summary of molecular dating analysis using (uncorrelated) relaxed lognormal clock.

Attribute	Analysis 1	Analysis 2	Analysis 3
**Dataset**	Mitochondrial	Nuclear	Concatenated
**Substitution model**	GTR+I+G	HKY+I+G	HKY+I+G
**MCMC runs (X10^7)**	150	150	200
**Overall ESS**	<200	>>200	<200
**Posterior**	-13117.02	-6161.38	-16360.55
**Prior**	69.56	-334.72	-11.17
**Likelihood**	-13186.6	-5826.66	-16349.37
**tmrca (AB/AN)**	4.89 ± 0.63	4.82 ± 0.95	1.24 ± 0.06
**tmrca (AC/AS)**	4.21 ± 0.66	3.05 ± 0.86	0.97 ± 0.07
**tmrca (Athene)**	7.22 ± 0.6	5.28 ± 0.44	1.91 ± 0.05
**tmrca (HB/AC/AS)**	7.21 ± 0.61	4.94 ± 0.65	1.78 ± 0.05
**tmrca (HB/AN/AB)**	6.63 ± 0.65	5.26 ± 0.47	1.91 ± 0.06
**tmrca (all) (Tytonidae / Strigidae)**	19.33 ± 2.17	45.1 ± 2.6	4.13 ± 0.14
**tmrca (Asioninae)**	11.52 ± 1.01	16.36 ± 1.24	2.22 ± 0.05
**tmrca (GR/GC)**	2.34 ± 0.47	0.98 ± 0.85	0.46 ± 0.07

**AB**: *A*. *brama*, **AN**: *A*. *noctua*, **AC**: *A*. *cunicularia*, **AS**: *A*. *superciliaris*, **HB**: *H*. *blewitti*, **GR**: *G*. *radiatum*, **GC**: *G*. *cuculoides*.

## Discussion

### 1. *Heteroglaux* as a synonym of *Athene*

Our analysis using mitochondrial, nuclear and concatenated datasets (Figs 2 and 3, and S4–S11 Figs) show that *H*. *blewitti* is nested within the *Athene* clade, rejecting the König *et al*. (1999) hypothesis that the species is nested within *Glaucidium* [23], and the Rasmussen & Collar (2013) hypothesis that *Heteroglaux* is a monotypic genus sister to *Athene* [16]. Our results also refute the Wolters (1975) hypothesis [26] that *H*. *blewitti* is sister to *A*. *brama*. *H*. *blewitti* and *A*. *brama* show similarity in lengths of tibiotarsus and ulna, and relatively shorter tarsometatarsi as compared to *A*. *noctua* to occupy arboreal niche [16]. Therefore, our results support the Rasmussen & Collar (2013) interpretation of the morphological similarities in *A*. *brama* and *H*. *blewitti* being either convergence of traits or plesiomorphies, further supported by the observation that a strong arboreal nature is absent in *A*. *noctua*. We find two contrasting results–*H*. *blewitti* as a sister clade, either to *Athene* from Madagascar and the Americas (concatenated and nuclear datasets, Figs 2 and 3, S3–S9 and S11 Figs) or Eurasian *Athene* (mitochondrial dataset, Fig 3 and S10 Fig), making the exact phylogenetic position of the species uncertain. Phylogenetic analyses with additional sampling of genetic markers, individuals per species, and distinct subspecies of *A*. *brama*, *A*. *noctua*, and *A*. *cunicularia* may help provide a better resolution. Based on our results, we propose that *Heteroglaux* is treated as a synonym of *Athene*, identifying *Heteroglaux blewitti* as *Athene [Heteroglaux] blewitti*.

### 2. Molecular dating and biogeography of *A*. *[H*.*] blewitti*

The overlapping dates of diversification of *A*. *[H*.*] blewitti* (4.3–5.7 Ma), *A*. *brama* / *A*. *noctua* split (3.9–5.8 Ma), and *A*. *cunicularia* / *A*. *superciliaris* split (2.2–3.9 Ma), based on the nuclear dataset (Table 1, analysis 2), indicate a rapid diversification of all three owlets in India, perhaps in response to Plio-Pleistocene climatic fluctuations. This diversification is from the same period as *A*. *inexpectanta* (3.6–5.3 Ma, Early Pliocene), the oldest fossil *Athene* owl from Africa. Pavia *et al*. (2014) argue that the genus *Athene* originated in Africa and had a much wider distribution than previously thought [64]. Given the rich island endemic *Athene* fossil records, from Late Pliocene of Palearctic [67], Nearctic [68–71], and Early Pleistocene of Palearctic [60, 72, 73], we speculate that *Athene* species might have undergone multiple diversifications and extinction events, possibly as a response to Plio-Pleistocene climate change, as observed in other groups such as the Western Ghats montane birds [7, 74].

Perhaps the Plio-Pleistocene climatic fluctuations and the subsequent retraction of evergreen forests [75, 76], during the Upper Sivalik time (1.6–5.1 Ma) of India, facilitated *A*. *brama* expansion into Peninsular India, while *A*. *noctua* expanded northward to a cooler climate. Recent studies have shown that *H*. *blewitti* occupies moderately dense dry deciduous forests, with intermittent open spaces [77, 78]. This peculiar choice of habitat influenced by climate, along with prey preference and ecological interactions with other similar-sized competitors might have restricted the range of *A*. *[H*.*] blewitti*. The species’ diurnal, ambushing predatory nature might be an adaptation to maximally utilize the available niche, given the presence of other co-distributed crepuscular and nocturnal owlets such as *A*. *brama* and *G*. *radiatum* in the same area. Further information on the dietary preference of *H*. *blewitti* and its ecological interactions with other species would help understand its adaptations. Nevertheless, our study provides another line of evidence to the role of climatic fluctuations in the diversification of Indian birds.

Our divergence estimates, based on the nuclear dataset (Table 1, analysis 2), are overlapping but more recent (15.1–17.6 Ma) than those derived by Fuchs *et al*. (2008) for *A*. *otus* / *O*. *leucotis* split (16.7–19.3 Ma) [66]. For the Tytonidae / Strigidae split, our divergence estimate of 42.5–47.7 Ma (Table 1, analysis 2) overlaps with Ericsson et al. (2006) estimate of 40–60 Ma [53], however, it also presents an underestimate when compared with other studies [54–56]. Our molecular dating analyses runs that included mitochondrial DNA (Table 1, analysis 1 and 3) did not converge despite 1.5 to 2 billion runs, perhaps due to the saturation of signal for these deep lineages.

### 3. Conservation implications

Genetic sampling of tropical birds is poor, especially in the Old World Tropics, thereby impacting, regional conservation needs [4, 79]. Although the new information on the phylogenetic status of *A*. [*H*.] *blewitti* does not directly impact the IUCN status of the species, its ranking in international conservation listings that use phylogenetic information may change. The Evolutionary Distinct and Globally Endangered (EDGE) listing will perhaps no longer carry the same evolutionary distinctness score for the species [79].

*A*. *[H*.*] blewitti* is a species of Central Indian old growth dry deciduous forests, occurring in protected as well as non-protected areas [11]. Across most of its range, it is also co-distributed with *A*. *brama*, a phylogenetically close relative based on this study. *A*. [*H*.] *blewitti* is under severe threat of habitat loss due to large-scale logging, timber harvesting, and land-use change [11, 77, 78]. *A*. *brama*, on the other hand, occurs in the vicinity of human habitation [19]. Although we detected no admixture between *A*. *[H*.*] blewitti* and *A*. *brama* (a mitochondrial genetic distance of 16 ± 1% indicative of low sharing of alleles) in this study, hybridization cannot be wholly ruled out. In the rapidly changing human-dominated landscape of the Central India, circumstances are similar to other owls such as Barred and Spotted Owls [80] and Northern and California Spotted Owls [81], where hybridization facilitated by anthropogenic activities, has led to numerous conservation challenges.

With this first molecular phylogenetic study of this Critically Endangered species, we demonstrate that crucial information can only be obtained through capture-based sampling that strengthens and supports ecological data collected through conventional methods. Capture-based genetic studies still do not find support from conservation managers in India [82, 83], but such studies are instrumental in providing vital information on taxonomy, evolutionary biogeography, and in identifying conservation units. Our study provides the first genetic dataset that needs to be followed up with further spatially explicit sampling that can be used for conservation prioritization.

The new information provided here will facilitate both the taxonomic revision of the *Athene* / *Heteroglaux* clade and highlight the need for studies predicting species responses to climate change.

## Supporting information

S1 Fig Distribution of few Palearctic and Oriental owlets as per Birdlife International (2015). *H*. *blewitti* is the only range-restricted, rare owlet among Indian owlets.
(TIF)

S2 Fig Map of sampling locations. (TIF)

S3 Fig Densitree representation, based on the Bayesian output of BEAST analysis, of the concatenated phylogenetic tree. **Blue line**: the consensus tree (primary hypothesis), **magenta line**: the next most popular tree after consensus (secondary hypothesis), **green lines**: tertiary hypotheses.
(TIF)

S4 Fig A Bayesian phylogenetic tree constructed using CYTB data. The species code used can be referred from S3 and S4 Tables. The nodal values show Bayesian posterior probability (PP) separated by Maximum Likelihood bootstrap support. **HB**: *H*. *blewitti*; **ASUP**: *A*. *superciliaris*; **ATHNB**: *A*. *brama*; **ANOCT**: *A*. *noctua*; **ACUN**: *A*. *cunicularia*; **GLRAD**: *G*. *radiatum* and **GCUCU**: *G*. *cuculoides*.
(TIF)

S5 Fig A Bayesian phylogenetic tree constructed using COI data. The species code used can be referred from S3 and S4 Tables. The nodal values show Bayesian posterior probability (PP) separated by Maximum Likelihood bootstrap support. **HB**: *H*. *blewitti*; **ASUP**: *A*. *superciliaris*; **ATHNB**: *A*. *brama*; **ANOCT**: *A*. *noctua*; **ACUN**: *A*. *cunicularia*; **GLRAD**: *G*. *radiatum* and **GCUCU**: *G*. *cuculoides*.
(TIF)

S6 Fig A Bayesian phylogenetic tree constructed using RAG-1 data. The species code used can be referred from S3 and S4 Tables. The nodal values show Bayesian posterior probability (PP) separated by Maximum Likelihood bootstrap support. **HB**: *H*. *blewitti*; **ASUP**: *A*. *superciliaris*; **ATHNB**: *A*. *brama*; **ANOCT**: *A*. *noctua*; **ACUN**: *A*. *cunicularia*; **GLRAD**: *G*. *radiatum* and **GCUCU**: *G*. *cuculoides*.
(TIF)

S7 Fig A Bayesian phylogenetic tree constructed using TGFB2 data. The species code used can be referred from S3 and S4 Tables. The nodal values show Bayesian posterior probability (PP) separated by Maximum Likelihood bootstrap support. **HB**: *H*. *blewitti*; **ASUP**: *A*. *superciliaris*; **ATHNB**: *A*. *brama*; **ANOCT**: *A*. *noctua*; **ACUN**: *A*. *cunicularia*; **GLRAD**: *G*. *radiatum* and **GCUCU**: *G*. *cuculoides*.
(TIF)

S8 Fig A Bayesian phylogenetic tree constructed using MYO data. The species code used can be referred from S3 and S4 Tables. The nodal values show Bayesian posterior probability (PP) separated by Maximum Likelihood bootstrap support. **HB**: *H*. *blewitti*; **ASUP**: *A*. *superciliaris*; **ATHNB**: *A*. *brama*; **ANOCT**: *A*. *noctua*; **ACUN**: *A*. *cunicularia*; **GLRAD**: *G*. *radiatum* and **GCUCU**: *G*. *cuculoides*.
(TIF)

S9 Fig A Bayesian phylogenetic tree constructed using LDH data. The species code used can be referred from S3 and S4 Tables. The nodal values show Bayesian posterior probability (PP) separated by Maximum Likelihood bootstrap support. **HB**: *H*. *blewitti*; **ASUP**: *A*. *superciliaris*; **ATHNB**: *A*. *brama*; **ACUN**: *A*. *cunicularia*; **GLRAD**: *G*. *radiatum* and **GCUCU**: *G*. *cuculoides*.
(TIF)

S10 Fig A Bayesian phylogenetic tree constructed using mitochondrial dataset (CYTB + COI). The species code used can be referred from the S4 Table. The nodal values show Bayesian posterior probability (PP) separated by Maximum Likelihood bootstrap support. **HB**: *H*. *blewitti*; **ASUP**: *A*. *superciliaris*; **ATHNB**: *A*. *brama*; **ANOCT**: *A*. *noctua*; **ACUN**: *A*. *cunicularia*; **GLRAD**: *G*. *radiatum* and **GCUCU**: *G*. *cuculoides*.
(TIF)

S11 Fig A Bayesian phylogenetic tree constructed using nuclear dataset (RAG-1 + TGFB2 + MYO). The species code used can be referred from the S4 Table. The nodal values show Bayesian posterior probability (PP) separated by Maximum Likelihood bootstrap support. **HB**: *H*. *blewitti*; **ASUP**: *A*. *superciliaris*; **ATHNB**: *A*. *brama*; **ANOCT**: *A*. *noctua*; **ACUN**: *A*. *cunicularia*; **GLRAD**: *G*. *radiatum* and **GCUCU**: *G*. *cuculoides*.
(TIF)

S1 Table Location data of the samples used. **BNHS**: Bombay Natural History Society museum; **JCT**: Jivdaya Charitable Trust (rescued bird); **CKV**: C.K. Vishnudas.
(DOCX)

S2 Table List of primers used. **Tm**: Optimal annealing temperature.
(DOCX)

S3 Table Provisional GenBank accession numbers of the sequences generated during the study. (DOCX)

S4 Table GenBank accession numbers of the sequences used in the current study. **NA**: Not available/Not used.
(DOCX)

S5 Table Best-fit partitioning scheme for genes used in the study. (DOCX)
