# Supplementary figures and images for: The critically endangered forest owlet Heteroglaux blewitti is nested within the currently recognized Athene clade: A century-old debate addressed
Source: PLoS One. 2018 Feb 5;13(2):e0192359. doi: 10.1371/journal.pone.0192359 (PMC5798823; doi:10.1371/journal.pone.0192359)

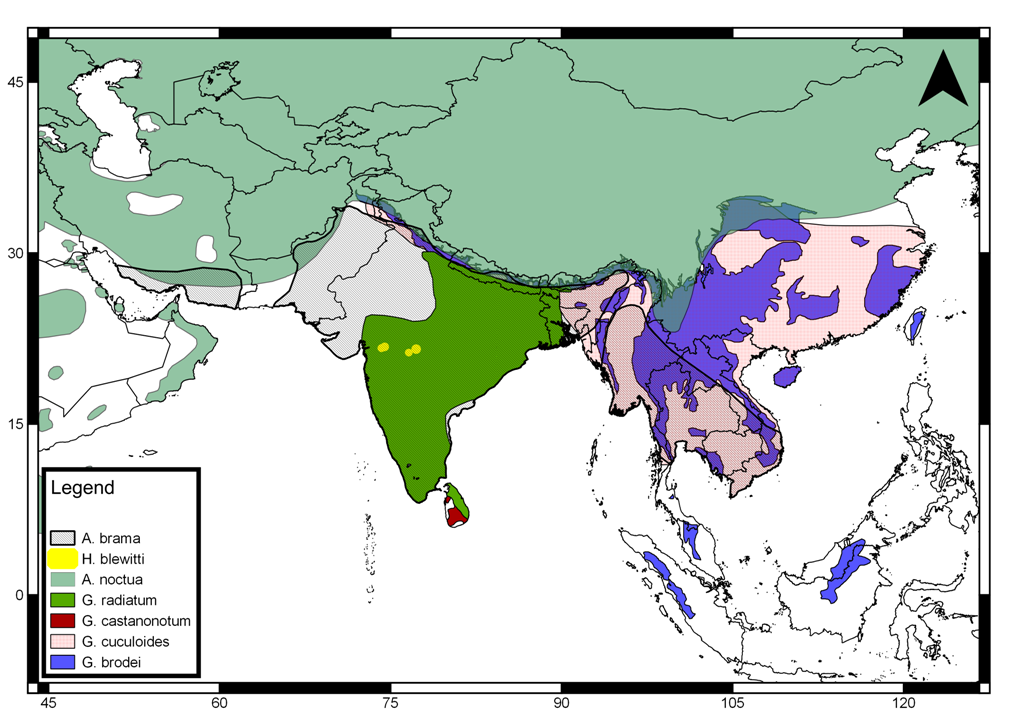

Supplement: S1 Fig — H. blewitti is the only range-restricted, rare owlet among Indian owlets. (TIF) [file pone.0192359.s001.tif]

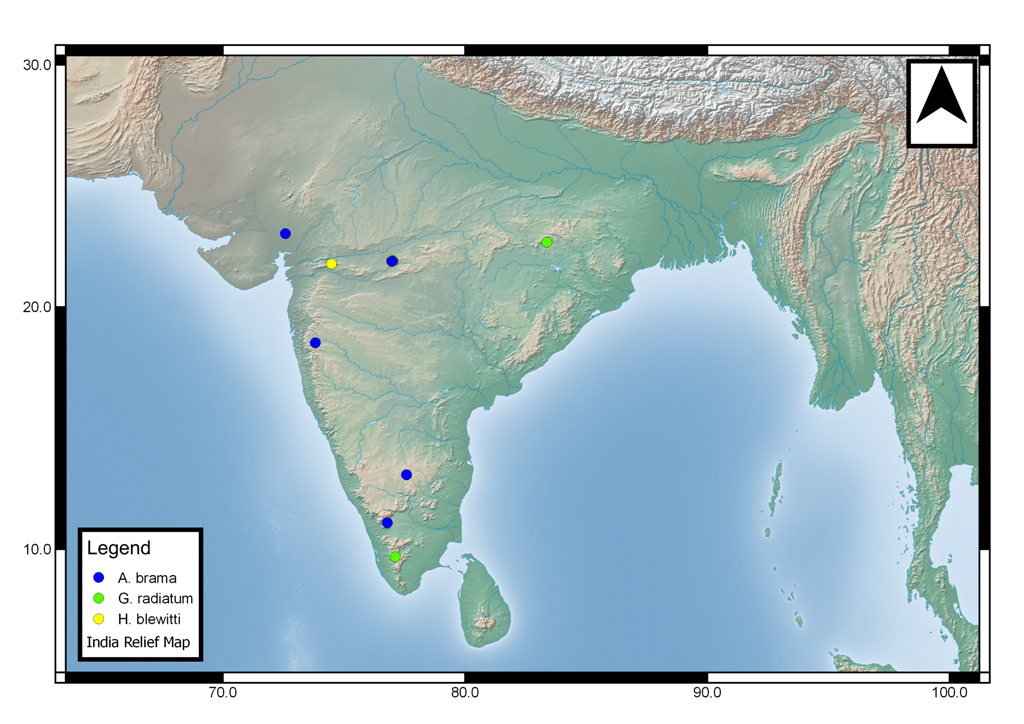

Supplement: S2 Fig — (TIF) [file pone.0192359.s002.tif]

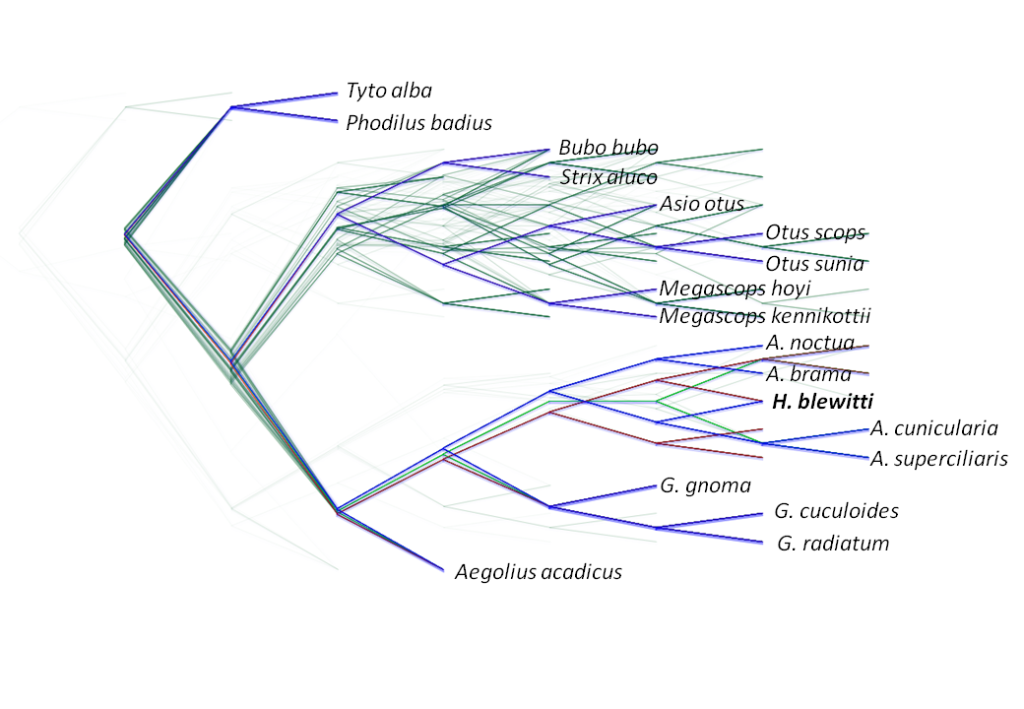

Supplement: S3 Fig — Blue line: the consensus tree (primary hypothesis), magenta line: the next most popular tree after consensus (secondary hypothesis), green lines: tertiary hypotheses. (TIF) [file pone.0192359.s003.tif]

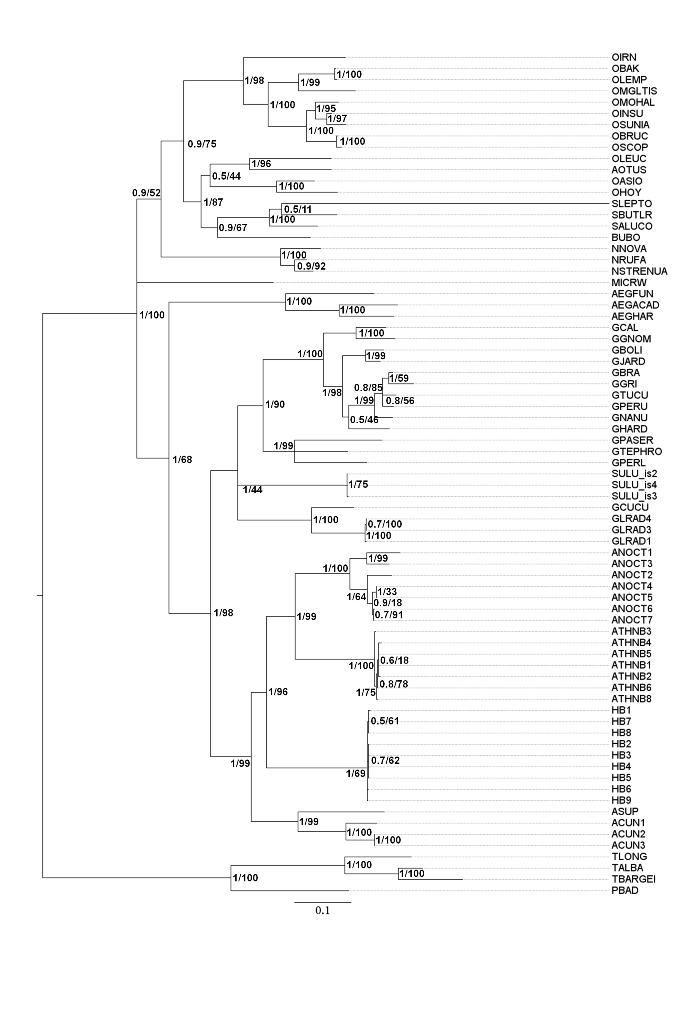

Supplement: S4 Fig — The species code used can be referred from S3 and S4 Tables. The nodal values show Bayesian posterior probability (PP) separated by Maximum Likelihood bootstrap support. HB: H. blewitti; ASUP: A. superciliaris; ATHNB: A. brama; ANOCT: A. noctua; ACUN: A. cunicularia; GLRAD: G. radiatum and GCUCU: G. cuculoides. (TIF) [file pone.0192359.s004.tif]

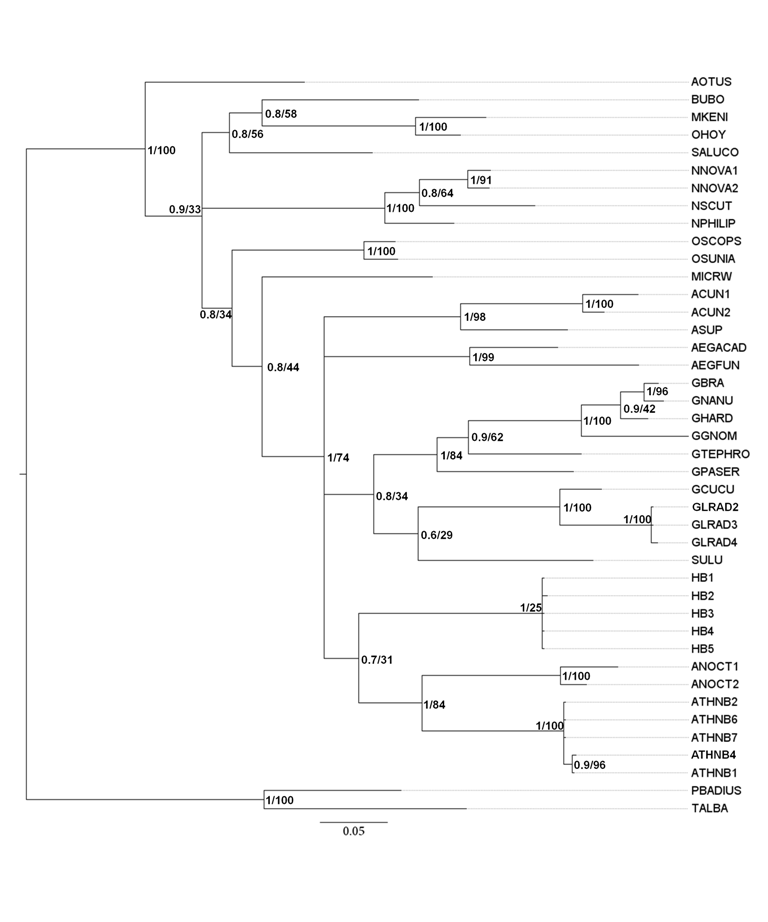

Supplement: S5 Fig — The species code used can be referred from S3 and S4 Tables. The nodal values show Bayesian posterior probability (PP) separated by Maximum Likelihood bootstrap support. HB: H. blewitti; ASUP: A. superciliaris; ATHNB: A. brama; ANOCT: A. noctua; ACUN: A. cunicularia; GLRAD: G. radiatum and GCUCU: G. cuculoides. (TIF) [file pone.0192359.s005.tif]

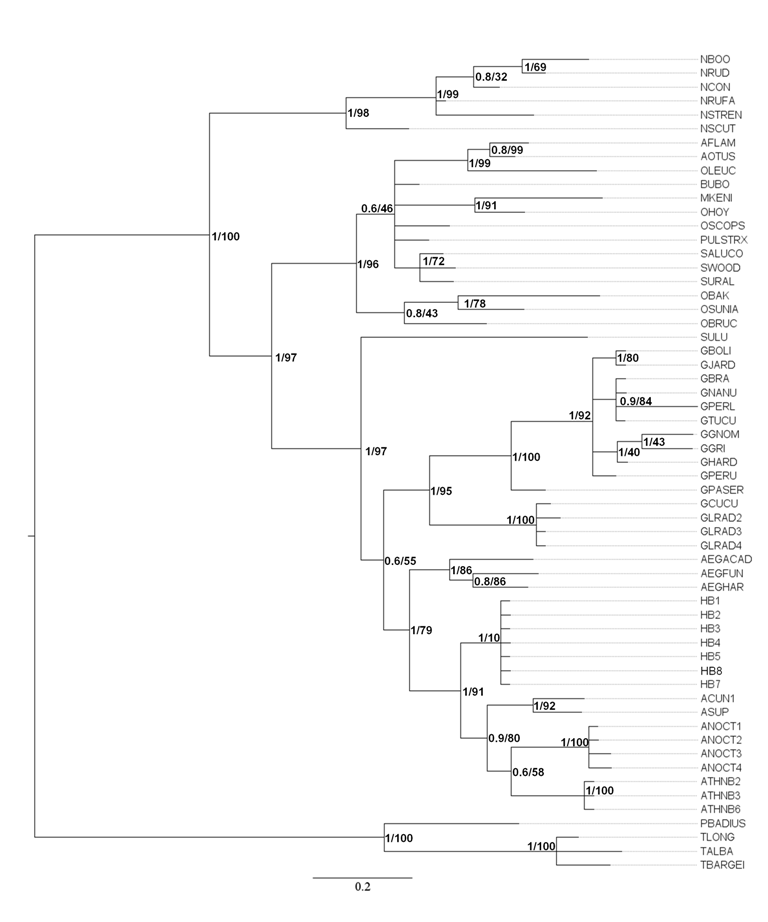

Supplement: S6 Fig — The species code used can be referred from S3 and S4 Tables. The nodal values show Bayesian posterior probability (PP) separated by Maximum Likelihood bootstrap support. HB: H. blewitti; ASUP: A. superciliaris; ATHNB: A. brama; ANOCT: A. noctua; ACUN: A. cunicularia; GLRAD: G. radiatum and GCUCU: G. cuculoides. (TIF) [file pone.0192359.s006.tif]

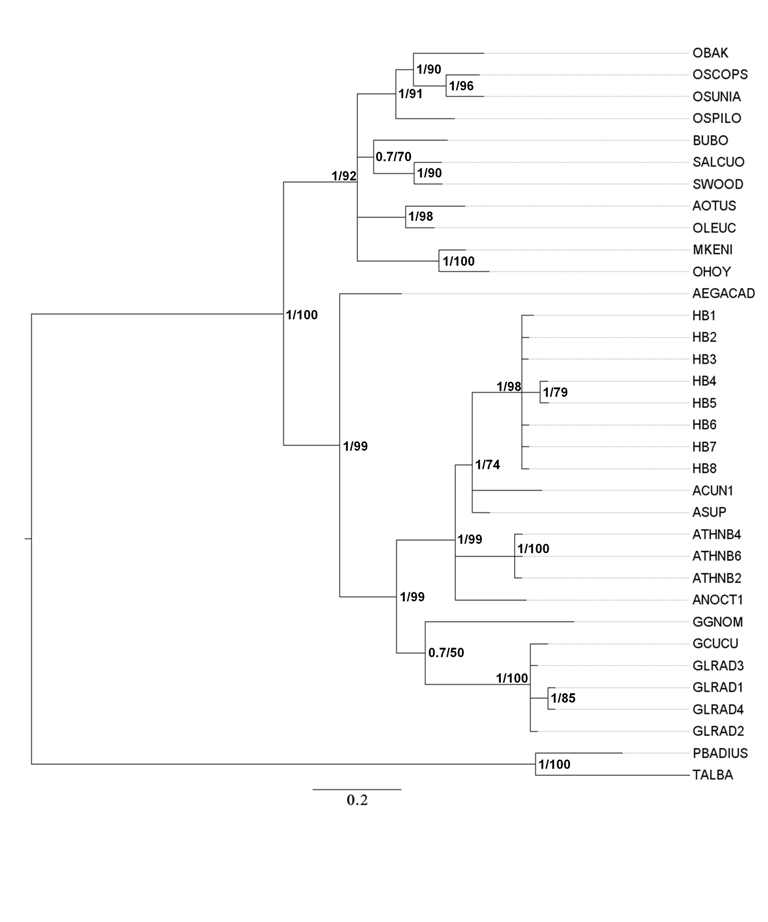

Supplement: S7 Fig — The species code used can be referred from S3 and S4 Tables. The nodal values show Bayesian posterior probability (PP) separated by Maximum Likelihood bootstrap support. HB: H. blewitti; ASUP: A. superciliaris; ATHNB: A. brama; ANOCT: A. noctua; ACUN: A. cunicularia; GLRAD: G. radiatum and GCUCU: G. cuculoides. (TIF) [file pone.0192359.s007.tif]

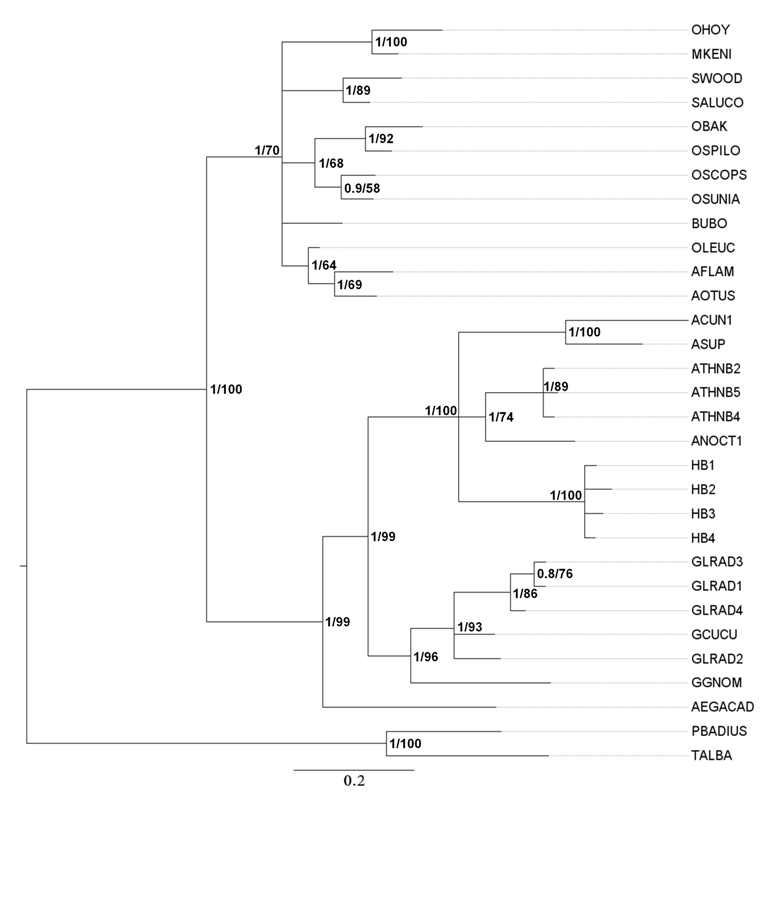

Supplement: S8 Fig — The species code used can be referred from S3 and S4 Tables. The nodal values show Bayesian posterior probability (PP) separated by Maximum Likelihood bootstrap support. HB: H. blewitti; ASUP: A. superciliaris; ATHNB: A. brama; ANOCT: A. noctua; ACUN: A. cunicularia; GLRAD: G. radiatum and GCUCU: G. cuculoides. (TIF) [file pone.0192359.s008.tif]

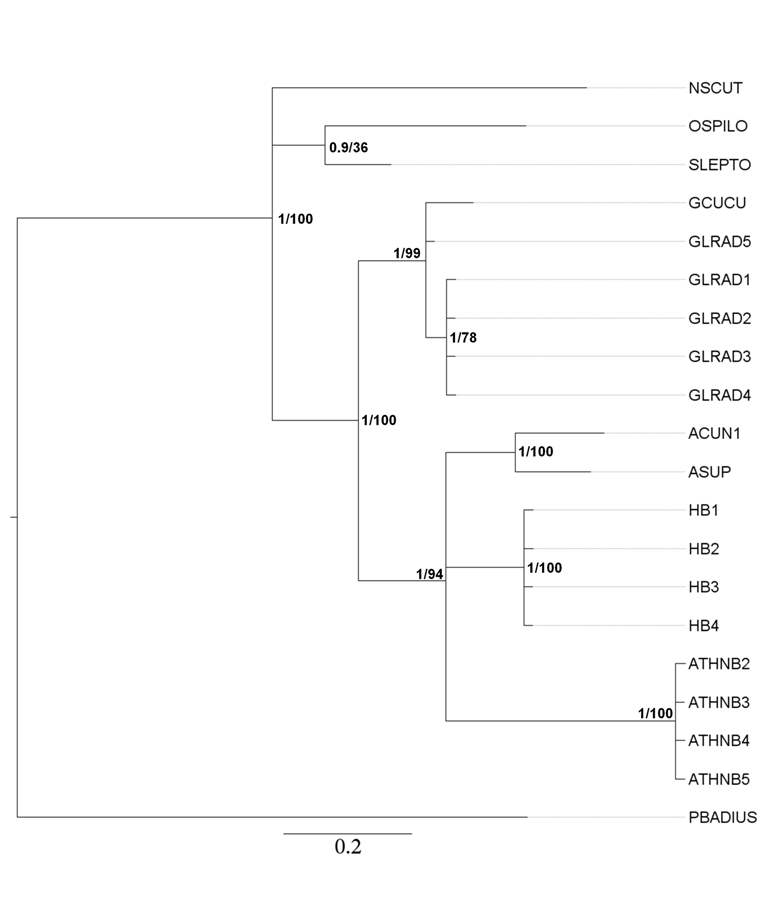

Supplement: S9 Fig — The species code used can be referred from S3 and S4 Tables. The nodal values show Bayesian posterior probability (PP) separated by Maximum Likelihood bootstrap support. HB: H. blewitti; ASUP: A. superciliaris; ATHNB: A. brama; ACUN: A. cunicularia; GLRAD: G. radiatum and GCUCU: G. cuculoides. (TIF) [file pone.0192359.s009.tif]

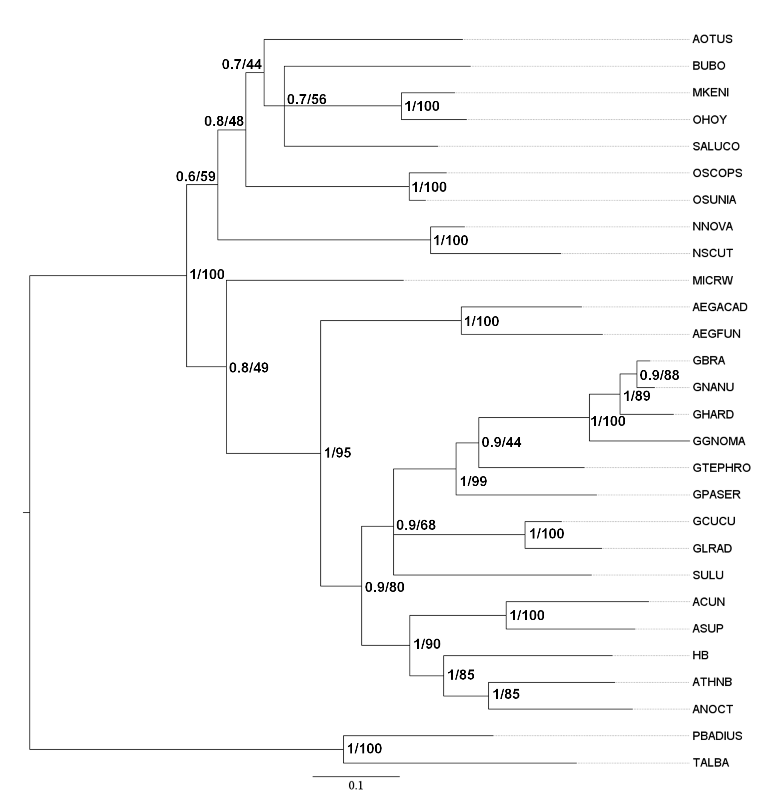

Supplement: S10 Fig — The species code used can be referred from the S4 Table. The nodal values show Bayesian posterior probability (PP) separated by Maximum Likelihood bootstrap support. HB: H. blewitti; ASUP: A. superciliaris; ATHNB: A. brama; ANOCT: A. noctua; ACUN: A. cunicularia; GLRAD: G. radiatum and GCUCU: G. cuculoides. (TIF) [file pone.0192359.s010.tif]

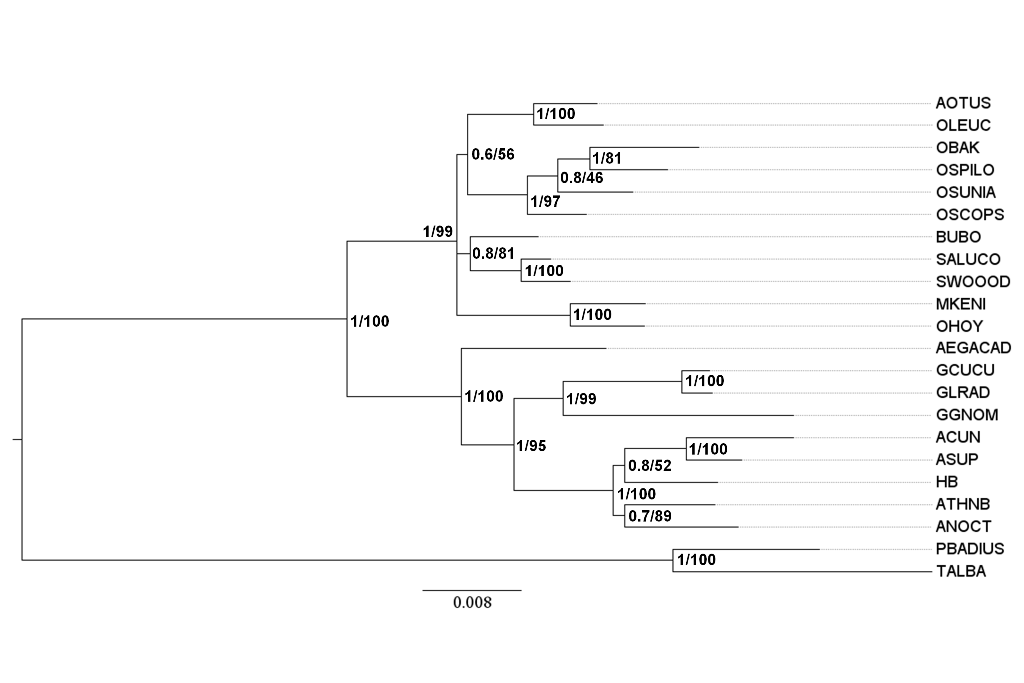

Supplement: S11 Fig — The species code used can be referred from the S4 Table. The nodal values show Bayesian posterior probability (PP) separated by Maximum Likelihood bootstrap support. HB: H. blewitti; ASUP: A. superciliaris; ATHNB: A. brama; ANOCT: A. noctua; ACUN: A. cunicularia; GLRAD: G. radiatum and GCUCU: G. cuculoides. (TIF) [file pone.0192359.s011.tif]
