# Supplementary material for: The critically endangered forest owlet Heteroglaux blewitti is nested within the currently recognized Athene clade: A century-old debate addressed
Source: PLoS One. 2018 Feb 5;13(2):e0192359. doi: 10.1371/journal.pone.0192359 (PMC5798823; doi:10.1371/journal.pone.0192359)
Supplement: S1 Table — BNHS: Bombay Natural History Society museum; JCT: Jivdaya Charitable Trust (rescued bird); CKV: C.K. Vishnudas. (DOCX) [file pone.0192359.s012.docx]

**Table 1.** Location data of the samples used. **BNHS:** Bombay Natural History Society museum; **JCT:** Jivdaya Charitable Trust (rescued bird); **CKV:** C.K. Vishnudas.

| **No.** | **Species** | **Sample code** | **Sample type** | **Locality** | **Collector** |
| --- | --- | --- | --- | --- | --- |
| 1 | *H. blewitti* | HB1 | feather | Auliya, Madhya Pradesh | PM |
| 2 | *H. blewitti* | HB2 | feather | Auliya, Madhya Pradesh | PM |
| 3 | *H. blewitti* | HB3 | feather | Auliya, Madhya Pradesh | PM |
| 4 | *H. blewitti* | HB4 | feather | Auliya, Madhya Pradesh | PM |
| 5 | *H. blewitti* | HB5 | feather | Auliya, Madhya Pradesh | PM |
| 6 | *H. blewitti* | HB6 | old feather | Toranmal, Maharashtra  Bombay Natural History Society Museum (BNHS 29004) | BNHS museum |
| 7 | *H. blewitti* | HB7 | old egg shell | Auliya, Madhya Pradesh | PM |
| 8 | *H. blewitti* | HB8 | egg shell | Auliya, Madhya Pradesh | PM |
| 9 | *A. brama* | ATHNB1 | feather | Coimbatore, Tamil Nadu | PK |
| 10 | *A. brama* | ATHNB2 | feather | Auliya, Madhya Pradesh | PK |
| 11 | *A. brama* | ATHNB3 | feather | Pune, Maharashtra | PK |
| 12 | *A. brama* | ATHNB4 | feather | Ahmadabad, Gujarat | JCT |
| 13 | *A. brama* | ATHNB5 | feather | Bangalore, Karnataka | PK |
| 14 | *A. brama* | ATHNB6 | feather | Bangalore, Karnataka | PK |
| 15 | *G. radiatum* | GLRAD1 | feather | Auliya, Madhya Pradesh | PK |
| 16 | *G. radiatum* | GLRAD2 | feather | Kerala | CKV |
| 17 | *G. radiatum* | GLRAD3 | blood | Rajapur, Chhattisgarh | PK |
| 18 | *G. radiatum* | GLRAD4 | blood | Rajapur, Chhattisgarh | PK |
| 19 | *A. superciliaris* | ASUP | museum tissue | Field Museum of Natural History (no. 384685, field no. SMG-8043) | SR |
