# Supplementary material for: The critically endangered forest owlet Heteroglaux blewitti is nested within the currently recognized Athene clade: A century-old debate addressed
Source: PLoS One. 2018 Feb 5;13(2):e0192359. doi: 10.1371/journal.pone.0192359 (PMC5798823; doi:10.1371/journal.pone.0192359)
Supplement: S2 Table — Tm: Optimal annealing temperature. (DOCX) [file pone.0192359.s013.docx]

**Table 2.** List of primers used. **Tm:** Optimal annealing temperature.

| **Primer pair sequence (5’ to 3’ direction)** | **amplicon size** | **Target gene** | **Tm** | **Reference** |
| --- | --- | --- | --- | --- |
| **L14851** - CCTACTTAGGATCATTCGCCCT  **Hb494** - TTGTCTACTGAGAATCCTCCTCA | 600 bp | CYTB | 53 | 39, 40 |
| **B5F** - ACCCTAGTAGAATGAGCCTGAGG  **H4A** - AAGTGGTAAGTCTTCAGTCTTTGGTTTACAAGACC | 700 bp | CYTB | 53 | 34, 39 |
| **COX 1F** - TTCTCCAACCACAAAGACATTGGCAC  **COX 1R** - ACGTGGGAGATAATTCCAAATCCTG | 600 bp | COI | 53 | 38 |
| **ABCX 1F** - CCTCTACCTAATCTTCGGCGCCTG  **ABCX 1R** - GATAAGAATATAAACTTCTGGGTGGCC | 650 bp | COI | 59 | This study |
| **OLTCX 1F** - CTGRGCAGGCATRGYWGGAACAGCCCT  **OLTCX 1R** - GAGGATGTANACTTCTGGGTGKCC | 650 bp | COI | 64 | This study |
| **R17F -** CCCTCCTGCTGGTATCCTTGCTT  **R20R**- CCATCTATAATTCCCACTTCTGT | 600 bp | RAG-1 |  | 35 |
| **R19F** - GTCACTGGGAGGCAGATCTTCCA  **R22R** - GAATGTTCTCAGGATGCCTCCCAT | 600 bp | RAG-1 | 63 | 35 |
| **TGFB2.5F** - GAAGCGTGCTCTAGATGCTG  **TGFB2.6R** - AGGCAGCAATTATCCTGCAC | 600 bp | TGFB2 | 55 | 36, 37 |
| **M1F** - CATGTGAGAGTTGGGCTT  **M2R** - AACTGAAGGGACACTCAA | 600 bp | MYO | 48 | 42 |
| **P5F** - GCTTGCTCTGGTTGAYGTTATGG  **P6R** - CACATTCCTCTGCACYAGGTTGAG | 600 bp | LDH | 56 | 41 |
