# Supplementary material for: The critically endangered forest owlet Heteroglaux blewitti is nested within the currently recognized Athene clade: A century-old debate addressed
Source: PLoS One. 2018 Feb 5;13(2):e0192359. doi: 10.1371/journal.pone.0192359 (PMC5798823; doi:10.1371/journal.pone.0192359)
Supplement: S3 Table — (DOCX) [file pone.0192359.s014.docx]

**Table 3.** **Provisional NCBI accession numbers of the sequences generated during the study.**

| Species | Code | CYTB | COI | RAG-1 | TGFB2 | MYO | LDH |
| --- | --- | --- | --- | --- | --- | --- | --- |
| *H. blewitti* | HB1 | MF871714 | MF871731 | MF871740 | MF871758 | MF871769 | MF871781 |
| *H. blewitti* | HB2 | MF871715 | MF871732 | MF871741 | MF871759 | MF871770 | MF871782 |
| *H. blewitti* | HB3 | MF871716 | MF871733 | MF871742 | MF871760 | MF871771 | MF871783 |
| *H. blewitti* | HB4 | MF871717 | MF871734 | MF871743 | MF871761 | MF871772 | MF871784 |
| *H. blewitti* | HB5 | MF871718 | MF871735 | MF871744 | MF871762 | - | - |
| *H. blewitti* | HB6 | MF871719 | - | - | MF871763 | - | - |
| *H. blewitti* | HB7 | MF871720 | - | MF871745 | MF871764 | - | - |
| *H. blewitti* | HB8 | MF871721 | - | MF871746 | MF871765 | - | - |
| *A. brama* | ATHNB1 | MF871710 | MF871726 | - | - | - | - |
| *A. brama* | ATHNB 2 | MF871709 | MF871722 | MF871736 | MF871750 | MF871766 | MF871777 |
| *A. brama* | ATHNB 3 | MF871708 | - | MF871737 | - | - | MF871778 |
| *A. brama* | ATHNB 4 | MF871705 | MF871723 | - | MF871751 | MF871767 | MF871779 |
| *A. brama* | ATHNB 5 | MF871706 | MF871725 | - | - | MF871768 | MF871780 |
| *A. brama* | ATHNB 6 | MF871707 | MF871724 | MF871738 | MF871752 | - | - |
| *G. radiatum* | GLRAD1 | MF871713 | - | - | MF871754 | MF871773 | MF871785 |
| *G. radiatum* | GLRAD 2 | - | MF871728 | MF871747 | MF871755 | - | MF871786 |
| *G. radiatum* | GLRAD 3 | MF871711 | MF871729 | MF871748 | MF871756 | MF871774 | MF871787 |
| *G. radiatum* | GLRAD 4 | MF871712 | MF871730 | MF871749 | MF871757 | MF871775 | MF871788 |
| *A. superciliaris* | ASUP | MF871704 | MF871727 | MF871739 | MF871753 | MF871776 | MF871789 |
