# Supplementary material for: The critically endangered forest owlet Heteroglaux blewitti is nested within the currently recognized Athene clade: A century-old debate addressed
Source: PLoS One. 2018 Feb 5;13(2):e0192359. doi: 10.1371/journal.pone.0192359 (PMC5798823; doi:10.1371/journal.pone.0192359)
Supplement: S4 Table — NA: Not available/Not used. (DOCX) [file pone.0192359.s015.docx]

**Table 4. GenBank accession numbers of the sequences used in the current study.** **NA:** Not available/Not used.

| **Species** | **Code** | **COI** | **CYTB** | **RAG-1** | **TGFB2** | **MYO** | **LDH** |
| --- | --- | --- | --- | --- | --- | --- | --- |
| *Aegolius acadicus* | AEGACAD | HM033213 | U89172 | EU348862 | EU600970 | EU601093 | NA |
| *Aegolius funereus* | AEGFUN | GU571227 | AJ004349 | EU348864 | NA | NA | NA |
| *Aegolius harrisii* | AEGHAR | NA | AJ003940 | EU348865 | NA | NA | NA |
| *Asio flammeus* | AFLAM | JF498831 | NA | EU348868 | NA | AY233366 | NA |
| *Asio otus* | AOTUS | GU571746 | AF082067 | EU348876 | EU600975 | EU601097 | NA |
| *Athene brama* | ATHNB7 | KF961185 | KF961185 | NA | NA | NA | NA |
| *Athene cunicularia* | ACUN1 | FJ027203 | AJ003944 | KJ455980 | EU737456 | EU740041 | KJ455225 |
| *Athene cunicularia* | ACUN2 | FJ027204 | KJ456202 | EU348871 | NA | NA | NA |
| *Athene cunicularia* | ACUN3 | FJ027206 | EU348965 | NA | NA | NA | NA |
| *Athene noctua* | ANOCT1 | JQ174101 | AJ003945 | EU348872 | EU600966 | EU601089 | NA |
| *Athene noctua* | ANOCT2 | JQ174102 | NA | NA | NA | NA | NA |
| *Athene noctua vidalii* | ANOCT3 | KF946603 | EU348967 | EU348875 | NA | NA | NA |
| *Athene noctua* | ANOCT4 | KF452069 | NA | NA | NA | NA | NA |
| *Athene noctua* | ANOCT5 | KF452076 | NA | NA | NA | NA | NA |
| *Athene noctua plumipes* | ANOCT6 | NA | EU348966 | EU348874 | NA | NA | NA |
| *Athene noctua lilith* | ANOCT7 | NA | NA | EU348873 | NA | NA | NA |
| *Bubo bubo* | BUBO | GU571764 | NA | NA | NA | EU601069 | NA |
| *Glaucidium bolivianum* | GBOLI | NA | AJ003975 | EU348894 | NA | NA | NA |
| *Glaucidium brasilianum* | GBRA | JN801686 | AY859400 | EU348895 | NA | NA | NA |
| *Glaucidium californicum* | GCAL | NA | AJ003993 | NA | NA | NA | NA |
| *Glaucidium cuculoides* | GCUCU | JQ174938 | KJ456288 | KJ456045 | EU600982 | EU601088 | KJ455255 |
| *Glaucidium gnoma* | GGNOM | AY666430 | AJ003994 | EU348897 | EU600972 | EU601094 | NA |
| *Glaucidium griseiceps* | GGRI | NA | AJ003995 | EU348898 | NA | NA | NA |
| *Glaucidium hardyi* | GHARD | JQ174941 | AJ003996 | EU348899 | NA | NA | NA |
| *Glaucidium jardinii* | GJARD | NA | AJ003998 | EU348900 | NA | NA | NA |
| *Glaucidium nanum* | GNANU | FJ027632 | AJ003999 | EU348901 | NA | NA | NA |
| *Glaucidium passerinum* | GPASER | GU571423 | AJ004000 | EU348902 | NA | NA | NA |
| *Glaucidium perlatum* | GPERL | NA | EU348979 | EU348903 | NA | NA | NA |
| *Glaucidium peruanum* | GPERU | NA | AJ004005 | EU348904 | NA | NA | NA |
| *Glaucidium tephronotum* | GTEPHRO | JQ174945 | AJ004006 | NA | NA | NA | NA |
| *Glaucidium tucumanum* | GTUCU | NA | AJ003992 | EU348905 | NA | NA | NA |
| *Megascops kennicottii* | MKENI | DQ433785 | NA | NA | NA | JF909608 | NA |
| *Micrathene whitneyi* | MICRW | DQ433022 | NA | NA | NA | NA | NA |
| *Ninox boobook* | NBOO | NA | NA | EU348914 | NA | NA | NA |
| *Ninox connivens* | NCON | NA | NA | EU348913 | NA | NA | NA |
| *Ninox novaeseelandiae* | NNOVA | NA | AJ004007 | NA | NA | NA | NA |
| *Ninox novaeseelandiae* | NNOVA1 | JQ175561 | NA | NA | NA | NA | NA |
| *Ninox novaeseelandiae* | NNOVA2 | NC_005932 | NA | NA | NA | NA | NA |
| *Ninox philippensis* | NPHIL | U83783 | NA | NA | NA | NA | NA |
| *Ninox rudolfi* | NRUD | NA | NA | EU348915 | NA | NA | NA |
| *Ninox rufa* | NRUFA | NA | EU348983 | EU348916 | NA | NA | NA |
| *Ninox scutulata* | NSCUT | AB843616 | AJ004008 | EU348917 | NA | NA | KJ455277 |
| *Ninox strenua* | NSTREN | NA | NA | EU348918 | NA | NA | NA |
| *Otus (Megascops) Asio* | OASIO | NA | DQ190845 | NA | NA | NA | NA |
| *Otus bakkamoena* | OBAK | NA | NA | NA | NA | EU601074 | NA |
| *Otus brucei* | OBRU | NA | EU348985 | EU348920 | NA | NA | NA |
| *Otus (Megascops) hoyi* | OHOY | FJ027786 | NA | NA | NA | EU601061 | NA |
| *Otus ireanae* | OIRE | NA | EU601113 | NA | NA | NA | NA |
| *Otus insularis* | OINSU | NA | EU601101 | NA | NA | NA | NA |
| *Otus lempiji* | OLEMP | NA | EU601112 | NA | NA | NA | NA |
| *Otus (Ptilopsis) leucotis* | OLEUC | NA | EU601120 | NA | NA | EU601085 | NA |
| *Otus megalotis* | OMGLTIS | U83779 | AJ004032 | EU348924 | NA | NA | NA |
| *Otus mohaliensis* | OMOHAL | NA | EU601121 | NA | NA | NA | NA |
| *Otus scops* | OSCOPS | JQ175647 | NA | NA | NA | EU601079 | NA |
| *Otus spilocephalus* | OSPIL | NA | NA | KJ456094 | EU600980 | EU601080 | NA |
| *Otus sunia* | OSUN | GQ482289 | NA | NA | NA | EU601081 | NA |
| *Phodilus badius* | PBADIUS | NC_023787 | NA | NA | NA | EU740015 | NA |
| *Pulsatrix perspilata* | PULSTRX | JQ176052 | NA | NA | NA | NA | NA |
| *Strix aluco* | SALUCO | GU572106 | NA | NA | NA | EU601070 | NA |
| *Strix leptogrammica* | SLEPTO | NA | NA | KJ456150 | NA | NA | KJ455291 |
| *Strix butleri* | SBUTLR | NA | EU348994 | NA | NA | NA | NA |
| *Strix uralensis* | SURAL | GU572109 | NA | NA | NA | NA | NA |
| *Strix woodfordii* | SWORD | NA | NA | NA | NA | EU601071 | NA |
| *Surnia ulula* | SULU | GU571640 | NA | EU348942 | NA | NA | NA |
| *Surnia ulula* | SULU_is2 | NA | AJ004067 | NA | NA | NA | NA |
| *Surnia ulula* | SULU_is3 | NA | AJ004068 | NA | NA | NA | NA |
| *Surnia ulula* | SULU_is4 | NA | AJ004069 | NA | NA | NA | NA |
| *Tyto alba* | TALBA | FJ028529 | FJ588458 | EU348946 | EU600971 | DQ881879 | NW_010001283 |
| *Tyto bargei* | TBARG | NA | EU349000 | EU348947 | NA | NA | NA |
| *Tyto longimembris* | TLONG | JQ287741 | EU349008 | EU348955 | NA | NA | NA |
