# Supplementary material for: The critically endangered forest owlet Heteroglaux blewitti is nested within the currently recognized Athene clade: A century-old debate addressed
Source: PLoS One. 2018 Feb 5;13(2):e0192359. doi: 10.1371/journal.pone.0192359 (PMC5798823; doi:10.1371/journal.pone.0192359)
Supplement: S5 Table — (DOCX) [file pone.0192359.s016.docx]

**Table 5.** **Best-fit partitioning scheme for genes used in the study.**

| **Gene** | **models for programs** | **Codon_1** | **Codon_2** | **Codon_3** |
| --- | --- | --- | --- | --- |
| CYTB | raxml | GTR+I+G | GTR+G | GTR+G |
|  | bayes | GTR+I+G | GTR+G | GTR+G |
|  | beast | GTR+I+G | GTR+G | GTR+G |
| COI | raxml | GTR+G | GTR+G | GTR+I+G |
|  | bayes | K80+G | GTR+G | GTR+I+G |
|  | beast | K80+I+G | GTR+G | GTR+I+G |
| RAG-1 | raxml | GTR+I+G | GTR+I+G | GTR+G |
|  | bayes | HKY+I | HKY+I | K80+G |
|  | beast | HKY+I | HKY+I | K80+G |
| LDH | raxml | GTR+G | | |
|  | bayes | HKY | | |
|  | beast | HKY | | |
| MYO | raxml | GTR+G | | |
|  | bayes | K80+G | | |
|  | beast | K80+G | | |
| TGFB2 | raxml | GTR+G | | |
|  | bayes | K80+G | | |
|  | beast | K80+G | | |
